# Targeted Delivery of Chlorin-e6-Loaded Carbon Nanotube-Based Nanobiocomposite to Cancer Stem Cells for Enhanced Photodynamic Therapy

**DOI:** 10.3390/pharmaceutics17040469

**Published:** 2025-04-03

**Authors:** Prabhavathi Sundaram, Sathish Sundar Dhilip Kumar, Heidi Abrahamse

**Affiliations:** Laser Research Centre, Faculty of Health Sciences, University of Johannesburg, Johannesburg 2028, South Africa; psundar@uj.ac.za (P.S.); sathishd@uj.ac.za (S.S.D.K.)

**Keywords:** hyaluronic acid, chlorin e6, colon cancer stem cells, photodynamic therapy, single-walled carbon nanotubes

## Abstract

**Background:** Globally, colorectal cancer (CRC) is the third-most diagnosed cancer among males and the second-most diagnosed cancer among females. In cancer, stem cells are a subset of neoplastic cells capable of tumorigenesis and exhibit properties like normal stem cells. Moreover, they are resistant to conventional cancer treatments and can repopulate the tumor following treatment. Cancer cells are stimulated to undergo apoptosis by photodynamic therapy (PDT), which involves a light source, a photosensitizer, and reactive oxygen species. **Methods:** In this study, colon cancer stem cells were isolated from colon cancer cells and characterized using flow cytometry and immunofluorescence techniques. To treat colon cancer stem cells (CCSCs), single-walled carbon nanotubes (SWCNTs) were coupled with hyaluronic acid (HA) and loaded with chlorin-e6 (Ce6). Nanobiocomposite toxicity was assessed using CCSCs with two fluences of 5 J/cm^2^ and 10 J/cm^2^. The cellular changes were observed at 24 and 48 h using microscopy, **Results:** LDH cytotoxicity assay, and cell death induction by annexin propidium iodide assay. An intracellular analysis of reactive oxygen species (ROS) detected oxidative stress within CCSCs. **Conclusions:** Overall, the results showed that the newly synthesized nanobiocomposite enhanced the ability of PDT to act as a photosensitizer carrier and induced cell death in CCSCs.

## 1. Introduction

Globally, large populations are affected by cancer, which has a high mortality rate. Cancer is mainly caused by several factors, including poor exercise habits, food habits, obesity, ultraviolet radiation, and genetic disorders [1]. The 5-year survival rate for patients diagnosed with colorectal cancer (CRC) is 15% if it has metastasized to other parts of the body, according to the American Cancer Society [2]. Metastasized CRC can be treated with chemotherapy, radiation therapy, targeted therapy, and immunotherapy [3]. It is possible to use these treatments individually or in combination to control cancer, slow its progression, and improve the quality of life for the patient [4]. Furthermore, clinical trials are available to explore new treatment options and advancements in the field. Photodynamic therapy (PDT) is a relatively new and emerging form of cancer treatment, involving three sources: laser light (light amplification by stimulated emission of radiation), photosensitizers (PS), and reactive oxygen species (ROS). When PS molecules are applied to target cells and then irradiated at a specific wavelength, they stimulate the production of singlet oxygen that causes necrosis or apoptosis in these cells [5]. Cancers such as bladder cancer, oesophageal cancer, skin cancer, and head and neck cancer can be treated with PDT. Additionally, it can be effective in the treatment of certain types of breast cancer, colorectal cancer, and lung cancer [6].

The term stem cell refers to pluripotent, multipotent, or unipotent, self-renewing cells which are generally classified into two types based on their characteristics and origin: embryonic stem cells and adult stem cells [7]. A cancer stem cell (CSC) has self-renewing and asymmetrical division properties, as well as the ability to form tumor cells with diverse phenotypes [8]. CSCs contribute to tumor heterogeneity, which is a key factor in cancer resistance and relapse. Cancer stem cells can produce a variety of tumor cell types, which can vary in terms of aggressiveness, response to treatment, and potential for metastasis [9]. The role of CSCs in tumor heterogeneity is crucial for developing more effective therapeutic strategies that target this population and prevent recurrence of tumors [10]. Several types of cancer have been found to contain CSCs, including CRC, breast, brain, melanoma, leukemia, and liver cancer [9,11]. Cancer stem cells represent a small population of cells that resist chemotherapy and then regenerate into tumors, leading to the failure of cancer treatment [12]. Immune checkpoint inhibitors have demonstrated significant promise in clinical trials by reactivating the immune system to target cancer cells. In addition to immune blockade, other emerging strategies in cancer therapy include monoclonal antibody-based targeting, antibody fragment-based targeting, and innovative theranostic approaches, all of which are advancing the field of precision oncology [13,14,15,16].

Single-walled carbon nanotubes (SWCNTs) are monolayered graphite sheets rolled into a tubular structure between 0.2 and 3 nm in diameter and are nanosized hollow cylindrical tubes. They are widely used in biomedical, nanocarrier, and imaging fields [17]. It is widely accepted that SWCNTs are the most effective nanocarriers in terms of their high surface area, biological distribution, biocompatibility, and ability to enter and exit the cell [18]. SWCNTs have shown promising results in delivering drugs to the brain and targeting specific brain regions, indicating their effectiveness as nanocarriers [19]. SWCNTs can effectively deliver genes into cells, leading to successful gene therapy [20]. These studies suggest that SWCNTs possess valuable characteristics that make them highly effective nanocarriers for biomedical applications.

In this study, we isolated CCSCs from Caco-2 colon cancer cells using the surface markers CD44, CD133, and CD24. These cells were cultured in ultra-low attachment flasks to maintain their stemness for further investigation. In our previous study, we outlined the synthesis procedure for single-walled carbon nanotubes (SWCNTs) functionalized with hyaluronic acid (HA) and loaded with Chlorine6 (Ce6) nanobiocomposite. The nanobiocomposite exhibited favorable physicochemical properties suitable for drug delivery applications [21,22]. Building upon these findings and the ongoing progression of our research, we subjected the synthesized nanocomposites to evaluation against isolated colon cancer stem cells (CCSCs). Treatment with photodynamic therapy (PDT) at 660 nm was administered to assess the combined effects of PDT and the nanobiocomposite on CCSCs.

## 2. Materials and Methods

### 2.1. Cell Culture

Colon cancer cell line Caco-2 was purchased from ATCC^®^, HTB-37™ and cultured in Dulbecco’s modified eagle’s medium (DMEM) (Sigma-Aldrich, Johannesburg, South Africa) supplemented with 10% (*v*/*v*) fetal bovine serum (FBS) (F9665-Sigma-Aldrich), 2 mM L-glutamate (G7513, Sigma-Aldrich, South Africa). Cultured cells were incubated at 37 °C in 5% CO_2_ and 85% humidity.

### 2.2. Isolation of Colon Cancer Stem Cells

Colon cancer stem cells were isolated from Caco-2 cell lines using magnetic-activated cell sorting MACS (Biocom Africa, Centurion, South Africa). CD133 (130-050-801, microbead kit, Miltenyi Biotec), CD44 (130-095-194, microbead kit, Biocom Africa, Centurion, South Africa), and CD24 (130-095-951, microbead kit, Miltenyi Biotec) markers are used to isolate the side population from the Caco-2 cell line. The positive selection of CD133+, CD44+, and CD24+ cells was separated using QuadroMACS™ protocol based on the principle of monoclonal antibody and specific antigen interactions.

### 2.3. Characterization of Isolated Colon Cancer Stem Cells

#### 2.3.1. Spheroid Formation

Isolated stem cells were incubated at 37 °C in 5% CO_2_ and 85% humidity in ultra-low attachment flasks in a complete stem cell medium. The spheroid formation images were captured under a light microscope at intervals during the observation of the flask.

#### 2.3.2. Immunofluorescence (IFL)

A direct and indirect immunofluorescence method was used to characterize the isolated CCSCs. CCSCs were visualized using primary antibody antibodies anti-human CD44 (MABF425, human/mouse, PE-Cy5, clone IM7, Sigma-Aldrich) CD133 (ZRB 1013, human/rabbit, clone F8, ZooAb, Merck, Modderfontein, South Africa) and CD24 (# 555426, human/mouse, clone ML5, BD Biosciences, Sandton, South Africa) conjugated with fluorochromes. CCSCs are directly labeled by primary antibodies directed at specific antigens and fluorochrome-conjugated secondary antibodies FITC (mouse anti-rabbit IgG, Sc 2359, Anatech Instruments (Pty) Ltd., Gauteng, South Africa), Cy5 (NB7602, Merck, Modderfontein, South Africa) directed against primary antibodies, followed by counterstaining with 300 nM DAPI, and the fluorescence was viewed under a live microscope. CCSCs were cultured on sterile coverslips placed in culture dishes at a concentration of 5 × 10^5^ cells in complete stem cell media.

#### 2.3.3. Flow Cytometry

To confirm the isolated side population, origin of CCSCs using fluorescently labeled antibodies conjugation was identified by flow cytometry. Primary antibodies mouse anti human CD44 (MABF425, human/mouse, PE-Cy5, clone IM7), CD133 (130-133-673, AC133, Vio^®^ Bright FITC) are fluorochrome conjugated so direct immunofluorescence method and CD24 (# 555426, human/mouse, clone ML5) indirect immunofluorescence method. For CD24 alone fluorochrome conjugated secondary antibodies Cy5 goat anti-mouse were used. Finally, the cell suspension was subjected to analysis using a flow cytometer, which detect the fluorescent probes on the cell surface. These results indicate the positive and negative selection of CD44, CD133, and CD24 markers on the surface of isolated CCSCs.

### 2.4. Synthesis of Nanobiocomposite

The purchased SWCNTs-COOH were purified using Nitric acid (HNO₃). Hyaluronic acid (HA) was dissolved in formamide, followed by the addition of EDC.HCl and NHS with stirring for 30 min. Trimethylamine (1 mL) was added dropwise in an ice bath, then stirred at RT for 2 h to activate HA. SWCNTs-COOH and HA-NH_2_ were mixed in formamide, sonicated, and reacted with EDC.HCl and NHS at RT for 15 min. Triethylamine (180 μL) was added dropwise in an ice bath and stirred at RT for 24 h. The reaction was cooled with acetone, centrifuged at 10,000 rpm for 15 min, dissolved in water, and dialyzed for 48 h. Ce6 was non-covalently bound to HA-SWCNTs via π π-interaction in DMF, sonicated for 10 min, and stirred overnight at RT [21].

### 2.5. Photodynamic Therapy on Isolated CCSCs

The red-light lasers were purchased from the National Laser Centre of South Africa (NLC). The laser output power was measured using a Field Mate Laser Power Meter (Coherent, 1098297) and a high-sensitivity thermopile sensor PM3 (Coherent, 1098336). As shown in Table 1, laser parameters were used for the study. A CCSCs was cultured in a Petri dish (3.4 cm) and treated with a nanobiocomposite before being irradiated with lasers.

#### 2.5.1. Morphology Studies

An inverted light microscope (CKX41, Olympus, Wirsam, Johannesburg, South Africa) equipped with a camera and getIT software version 5.2 was used to visualize the morphology of the treated cells. The cells were divided into three groups: group 1: cells alone and cells with SWCNTs, Ce6 and SWCNTs-HA-Ce6 without treatment; group 2: cells alone and cells with SWCNTs, Ce6 and SWCNTs-HA-Ce6 with treatment of fluence 5 J/cm^2^; group 3: cells alone and cells with SWCNTs, Ce6 and SWCNTs-HA-Ce6 with treatment of fluence 10 J/cm^2^. An IC50 established at 2.56 µg/mL for the nanobiocomposite was used throughout this study. A morphology analysis of each plate was performed after PDT at 0 h, 24 h, and 48 h. 

#### 2.5.2. Cytotoxicity Assay Using Lactate Dehydrogenase (LDH) Assay

The cytotoxicity range of synthesized nanbiocomposite under PDT treatment was measured using LDH assay kit (CytoTox 96^®^ Non-Radioactive Cytotoxicity colorimetric assay (G1780, Anatech Instruments (Pty) Ltd., Gauteng, South Africa). The PDT-treated cells were incubated for 24 h and 48 h at 37 °C in 5% CO_2_ and 85% humidity, then the 50 µL of medium from cells and 50 µL of reconstituted reagent mixed in flat bottom 96 well-clear polystyrene microplate (CLS3370, Corning^®^, Merck, Modderfontein, South Africa). The microplate was incubated for 30 min at room temperature in the wave motion mixer, and the compound was measured using a VICTOR3 multilabel plate counter.

#### 2.5.3. Flow Cytometry Annexin V–Fluorescein Isothiocyanate (FITC) and Propidium Iodide (PI)

The cellular apoptosis of synthesized nanbiocomposite under PDT treatment was analyzed using FITC Annexin V Apoptosis detection kit I (556547, BD Biosciences, Sandton, South Africa). The treated cells were rinsed with HBSS and detached using TrypLE express, and cell suspension was transferred into flow cytometry tubes and resuspended with 1X binding buffer. 5 µL of Annexin V-FITC and PI reagents were added into the cell suspension, mixed thoroughly, and incubated for 10 min at room temperature under dark conditions. Finally, the C6 flow cytometric analysis was performed to detect the FITC green and PI red fluorescence, respectively.

#### 2.5.4. Intracellular Reactive Oxygen Species Detection

The production of ROS of synthesized nanbiocomposite under PDT treatment was measured using fluorometric intracellular ROS kit (MAK142, Sigma-Aldrich). CCSCs were cultured on sterile coverslips placed in culture dishes, at a concentration of 5 × 10^5^ cells in complete stem cell media. After the attachment of cells, the newly synthesized nanbiocomposite was incorporated into the cells and irradiated at 660 nm and incubated for 1 h at 37 °C in 5% CO_2_ and 85% humidity. Cells were rinsed with HBSS and ROS master mix 100 µL was added into the cells incubated for 30 min at 37 °C. After incubation time, cells were washed with PBS, and 300 nM DAPI was added into the cells incubated for 20 min at 37 °C. The coverslip was mounted on the glass slide using a floor mount and visualized using a live cell microscope.

### 2.6. Statistical Analysis

The results were obtained, processed for graphic presentation, and data analysis was performed with Origin Pro 8 SRO (v8.0724) and BD CSampler™ software (version 227.4). All experiments were performed in triplicate to monitor the reproducibility of the results, and all data are expressed as the mean ± standard deviation. Differences between groups were determined using the one-tailed Student’s *t*-test.

## 3. Results and Discussion

### 3.1. Isolation and Characterization of CCSCs: Morphology, Flow Cytometry, Immunofluorescence

#### 3.1.1. Spheroid Formation of Isolated CCSCs

**Figure 1:**
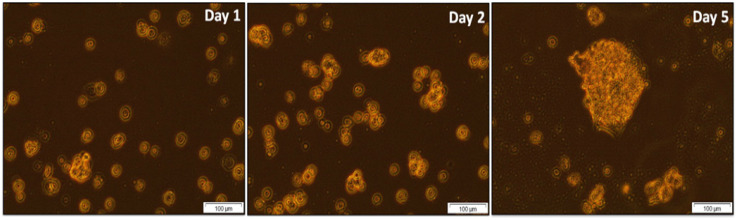
Microscopic images of isolated CCSCs forming spheroid colonies captured on Day 1, 2, and 5. Scale bar 100 µm.

CCSCs were cultured using a complete stem cell medium, and the cultured cells could self-renew stem cells and CSCs. It was found that the cultured cells grew very slowly in an ultra-low attachment flask. Figure 1 shows the morphological characteristics of the cells after one, two, and five days under an inverted light microscope. The images demonstrated the spheroid formation of isolated CCSCs using CD133, CD44, and CD24 markers. These findings strongly suggest that isolated cells in colon cancer cell lines Caco-2 possess features characteristic of CSCs. A study conducted identified the isolated side population cells from Caco-2 cells as colon cancer stem cells, which proliferated as mammospheres in an in vitro model of colon cancer. A secondary mammosphere could be formed by enzymatically degrading primary mammospheres into single cell suspension [23].

#### 3.1.2. Flow Cytometry Analysis

**Figure 2:**
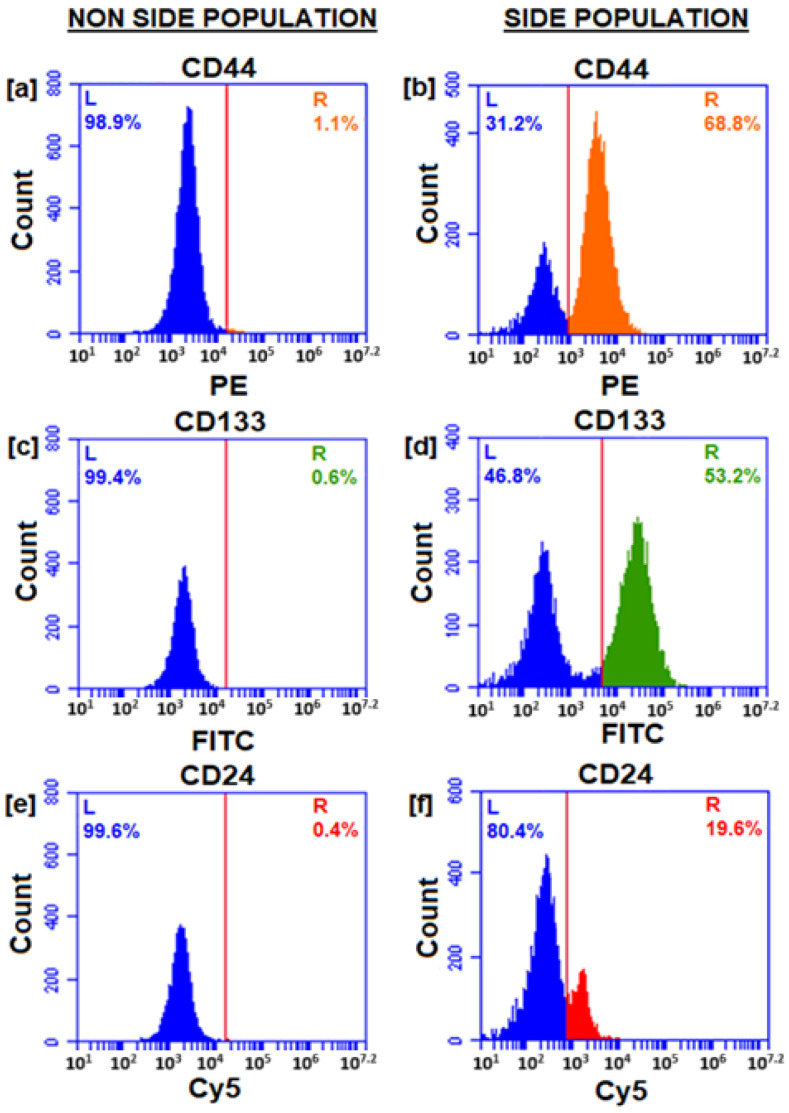
Expression of CD44, CD133, and CD24 surface markers as observed using flow cytometry. [(**a**,**c**,**e**)-Non-side population cells and (**b**,**d**,**f**)-side population cells].

Isolated cells express cell surface markers CD44 tagged with phycoerythrin (PE) in the yellow-orange region of the visible spectrum, and fluorescence was detected using FL-2 with a 585/40 filter, a 488 nm laser, PE maximum excitation at 565 nm, emission at 573 nm. In Figure 2, it is evident that CD 44 has a high expression level of approximately 68.8% in isolated CCSCs. The CD 133 cells tagged with FITC can be observed in the green region of the visible spectrum. The fluorescence was detected using FL-1 with a 533/30 filter, a 488 nm laser, the maximum excitation wavelength of FITC is 494 nm, and the maximum emission wavelength is 518 nm. Isolated CCSCs showed a high level of CD 133 expression, with about 53.2% being detected.

Cy5 tagged CD 24 shows fluorescence in the red region of the visible spectrum and was detected by FL-3 using a 670 LP filter and a 488 nm laser. The maximum excitation wavelength of Cy5 was 650 nm, while its emission wavelength was 670 nm. On the isolated CCSCs, CD 24 markers were observed in about 19.6% of the cells. A non-side population of cells tagged with CD44-PE, CD133-FITC, and CD24-Cy5 was analyzed. The results indicated that no positive cells had been detected. Cells isolated using magnetic beads are completely attracted to the positive cells, and then the negative cells are collected during the washing process. The results of this study confirm the successful isolation of CCSCs from Caco-2 cells, and the combination of three markers indicates that isolated CCSCs are more tumorigenic than those that are not.

#### 3.1.3. Immunofluorescence

An immunofluorescence technique involves using fluorochrome-attached antibodies to visualize the interaction between the antigen–antibody and the target antigen within the cell under a fluorescence microscope to detect specific antibody binding to the target antigen. There are two types of immunofluorescence techniques: direct and indirect. In the direct method, a primary antibody is linked to a fluorophore to target a specific antigen. The indirect method involves the use of two antibodies, primary and secondary. The primary antibody targets the antigen within the cell, and the secondary antibody recognizes the primary antibody and binds to it [24]. We used both indirect and direct immunofluorescence in this study.

**Figure 3:**
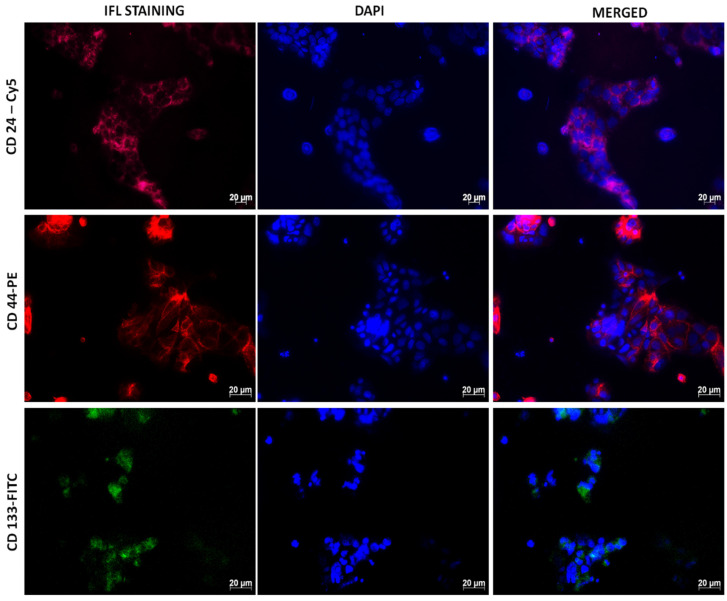
Fluorescent antigenic detection of the surface marker CD24, CD44, and CD133 IFL staining of the isolated side population of CCSCs. DAPI nuclei counter staining. Scale bar 20 µm.

We cultured the isolated CCSCs using a complete stem cell medium. The adherent culture of isolated CCSCs showed moderate expression of CD133, CD44, and CD24 markers for immunofluorescence. CCSCs nuclei were counter-stained using DAPI, and the markers CD133-FITC, CD44-PE, and CD24-Cy5 were detected directly and indirectly by immunofluorescence techniques (Figure 3). The CD133 markers are also known as Promini-1 (PROM1), a transmembrane glycoprotein that was mainly found in the plasma membrane and is overexpressed in CSCs. CD133 has been reported to be an independent marker for prognosis and chemoresistance in colon cancer in many studies [25,26]. The CD44 and CD24 markers have been widely used or proposed for identifying CSCs [27,28]. It is known that hyaluronan targets CD44 receptors on cancer cell surfaces and contributes to the spread of hemorrhage when it is in contact with L- or P-selectins [29]. CCSCs isolated from Caco-2 cells displayed the expression of CD133, CD44, and CD24 in the presence of fluorescence Cy5, PE, and FITC. 

### 3.2. Targeted Delivery of SWCNTs-HA-Ce6 on CCSCs

**Figure 4:**
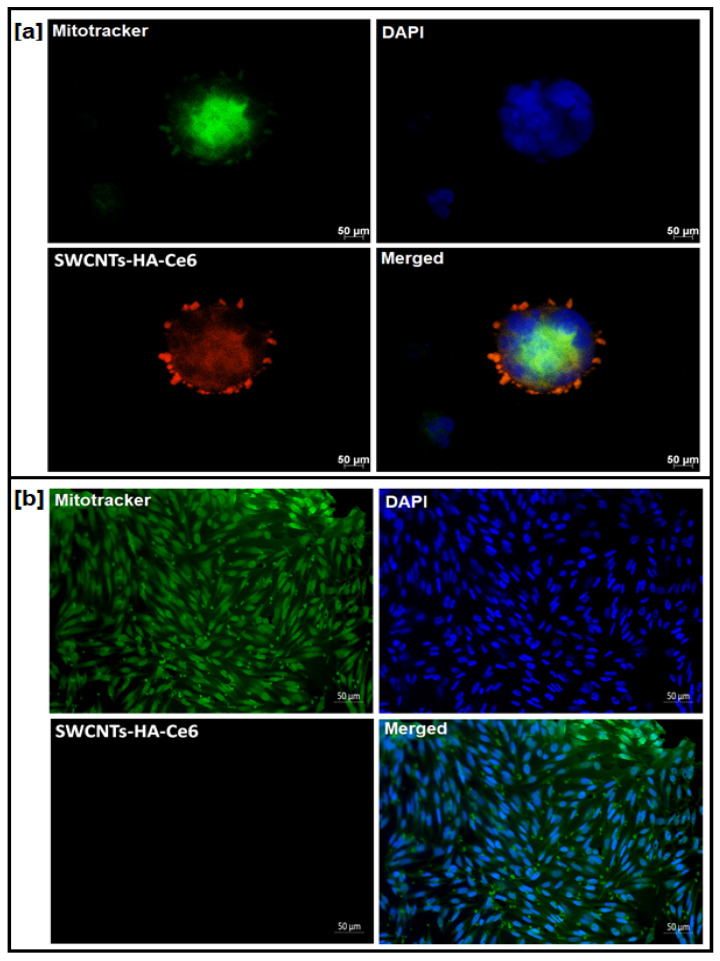
Targeted delivery of SWCNTs-HA-Ce6. (**a**) Isolated CCSCs (CD44 receptor positive cells) and (**b**) SK-UT-1 cells (CD44 receptor negative cells). Scale bar 50 µm.

A nanobiocomposite was synthesized with HA to target the CD44 receptors on cell surfaces, which are overexpressed in CCSCs. In this study, isolated CCSCs were cultured on sterile glass slides with SWCNTs-HA-Ce6 added and SK-UT-1 cells served as a negative control (CD44 receptor negative cells). We incubated the cells for 4 h at 37 °C in 5% CO_2_. CCSCs and SK-UT-1 cells were used to study the cellular imaging and targeting efficiency of SWCNTs-HA-Ce6 towards the CD44 receptor. The imaging of SWCNTs-HA-Ce6 was conducted in three channels (Mitotracker, DAPI, and SWCNTs-HA-Ce6), and the images were merged to determine the targeting efficiency of the SWCNTs-HA-Ce6. Figure 4 illustrates the results of this study.

The study conducted in CCSCs (Figure 4a) showed that SWCNTs-HA-Ce6 channels induced the fluorescence intensity and cellular uptake of Ce6. Furthermore, the HA functionalized SWCNTs-HA-Ce6 facilitates the targeting of SWCNTs-HA-Ce6 onto CD44 positive cells. Figure 4a revealed the presence of fluorescent SWCNTs-HA-Ce6 molecules in the mitochondrial region. There is no prominent update of SWCNTs-HA-Ce6 in SK-UT-1 cells due to the absence of the CD44 cell surface receptor (Figure 4b). SWCNTs-HA-Ce6 molecules are mainly located in the mitochondrial region CCSCs, which result in the destruction of spheroid or clumps of cells through laser energy [30]. Moreover, the HA functionalized SWCNTs-HA-Ce6 facilitates SWCNTs-HA-Ce6’s ability to target CD44 positive cells. It was concluded from this study that the synthesized SWCNTs-HA-Ce6 might be used as a potential nanomaterial in cancer therapy to target CCSCs.

### 3.3. Photodynamic Therapy Effect on Isolated CCSCs Using SWCNTs-HA-Ce6

#### 3.3.1. Cell Morphology Study

**Figure 5:**
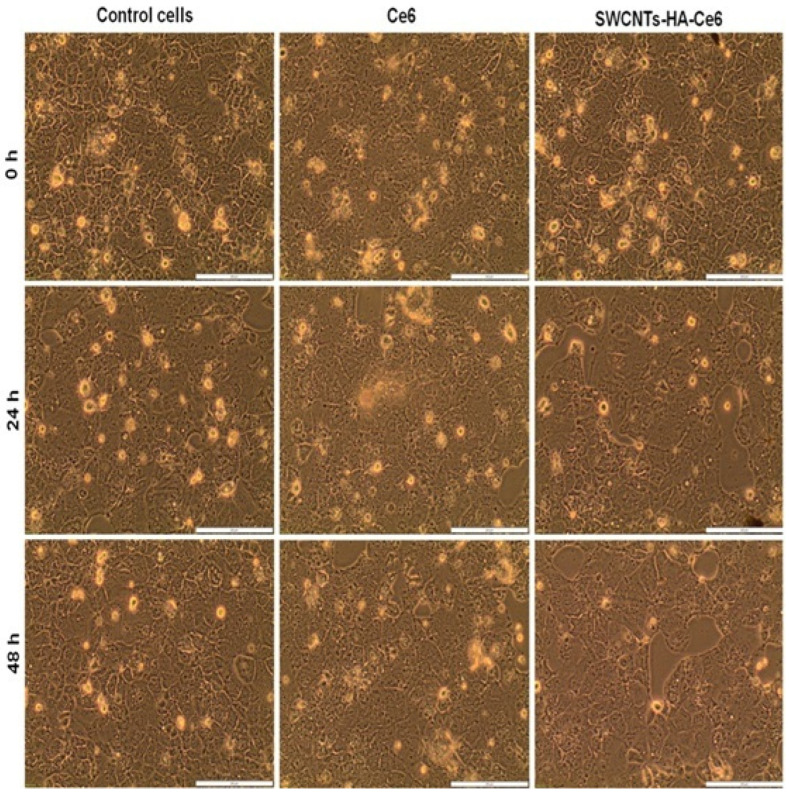
Morphology analysis using microscopic images of treated and untreated CCSCs at 0 h, 24 h and 48 h of laser irradiated at 660 nm, 0 J/cm^2^. Scale bar 200 µm.

**Figure 6:**
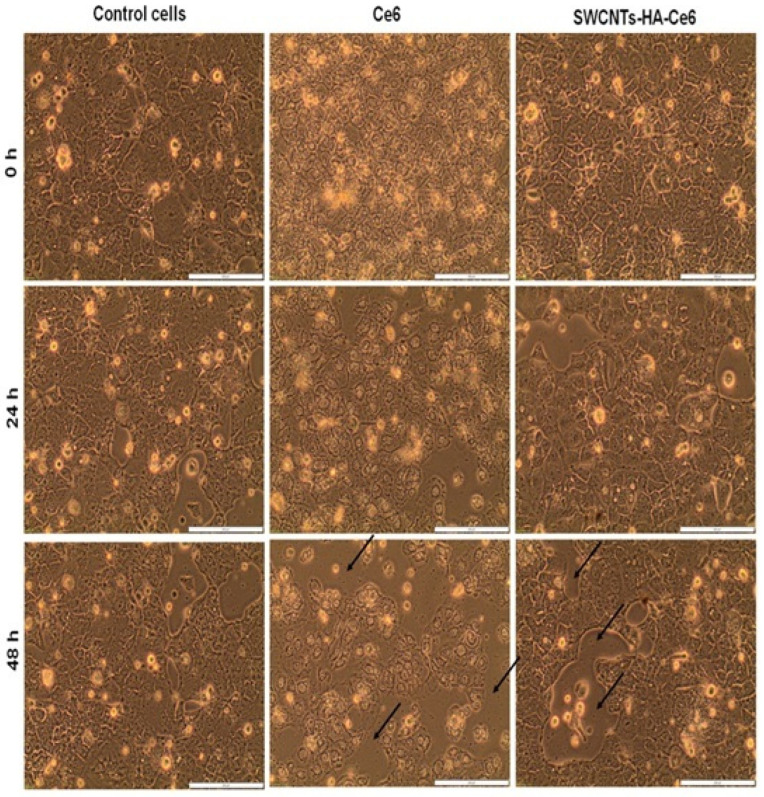
Morphology analysis using microscopic images of treated and untreated CCSCs at 0 h, 24 h and 48 h of laser irradiated at 660 nm, 5 J/cm^2^. Scale bar 200 µm.

**Figure 7:**
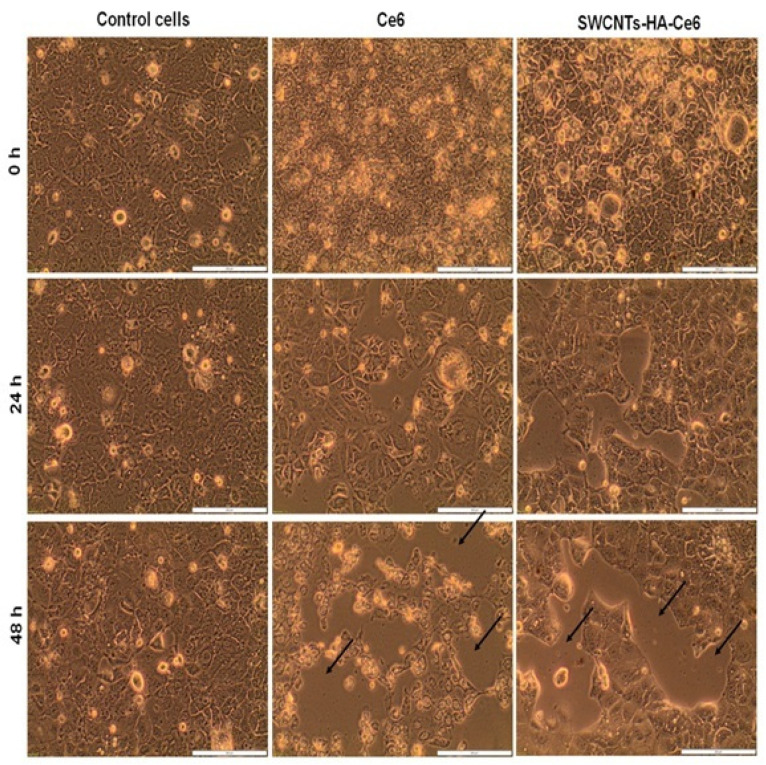
Morphology analysis using microscopic images of treated and untreated CCSCs at 0 h, 24 h and 48 h of laser irradiated at 660 nm, 10 J/cm^2^. Scale bar 200 µm.

Following the isolation of CCSCs from Caco-2 cells, the cells were cultured on Petri dishes at a density of 4 × 10^5^ cells. After reaching confluency, they were treated with laser irradiation at 660 nm fluences of 5 and 10 J/cm^2^. We observed the morphology of treated and untreated cells for 0 h, 24 h, and 48 h using an inverted light microscope, and our results are presented in Figure 5, Figure 6 and Figure 7. A preliminary assessment of cellular viability and death was conducted using the morphology of the cells captured under a microscope. SWCNTs-HA-Ce6 treated and laser irradiated (5 J/cm^2^) cells showed significant changes in cellular morphology at 48 h (Figure 6). A similar pattern of morphological changes and increased cell death was observed in SWCNTs-HA-Ce6-treated and laser-irradiated (10 J/cm^2^) cells at 48 h, as shown in Figure 7.

#### 3.3.2. LDH Cytotoxicity Assay

**Figure 8:**
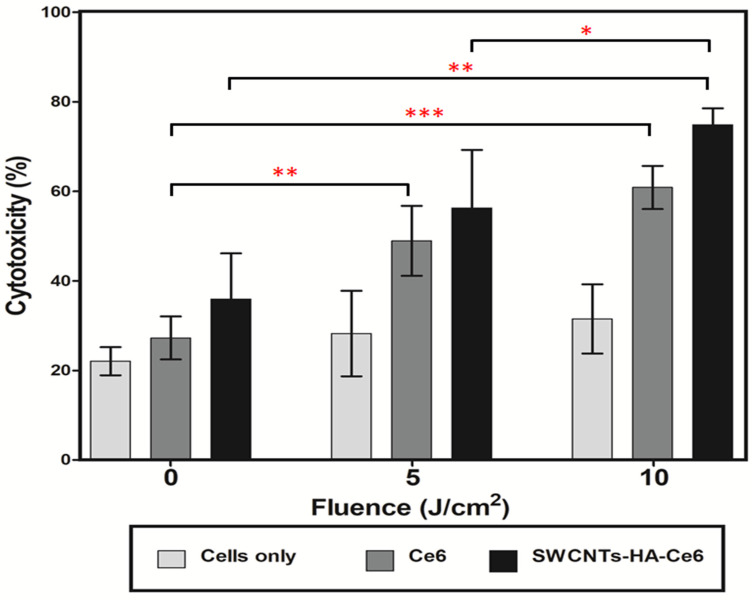
The cytotoxicity effects of Ce6, SWCNTs-HA-Ce6 on CCSCs, laser irradiated at 660 nm of fluence 0, 5, and 10 J/cm^2^ incubated for 24 h and determined cytotoxicity by LDH assay. Significance is shown as * *p* < 0.05; ** *p* < 0.01; *** *p* < 0.001.

**Figure 9:**
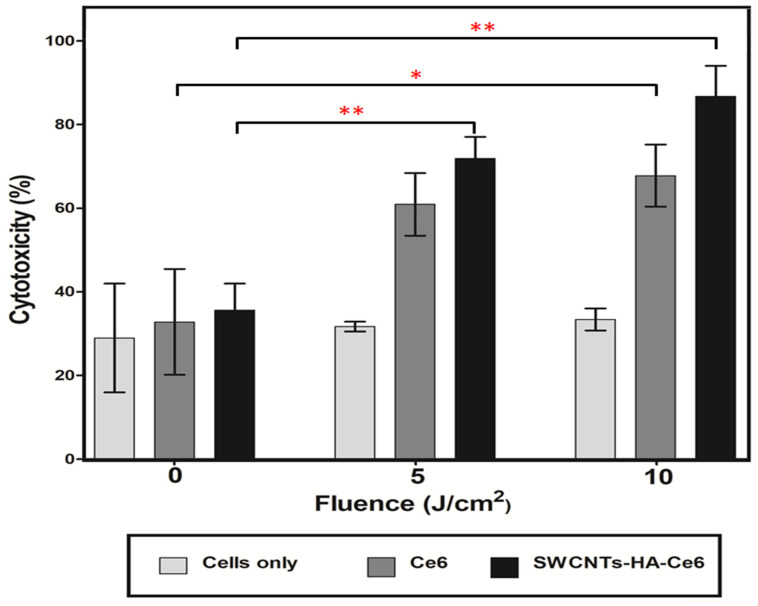
The cytotoxicity effects of Ce6, SWCNTs-HA-Ce6 on CCSCs, laser irradiated at 660 nm of fluence 0, 5, and 10 J/cm^2^ incubated for 48 h and determined cytotoxicity by LDH assay. Significance is shown as * *p* < 0.05; ** *p* < 0.01.

A LDH cytotoxicity assay kit was used to analyze the cytotoxicity of SWCNTs-Ce6-HA. The isolated CCSCs were incubated with Ce6 and SWCNTs-Ce6-HA, and then irradiated at 660 nm of laser fluence 0, 5, and 10 J/cm^2^. Following PDT treatment, the damaged cancer cell released LDH enzyme into the culture media, which were analyzed using LDH cytotoxicity assay kits. Untreated cells were used as a control. Figure 8 and Figure 9 illustrate the results of the assay. The 24 h cytotoxicity assay results are shown in Figure 8; the observed results revealed that the non-irradiated 0 J/cm^2^ showed lower percentage of cytotoxic behavior for Ce6 (27.25%) and SWCNTs-HA-Ce6 (35.94%).

SWCNTs-HA-Ce6 treated cells showed 56.3% cytotoxicity when laser irradiated at 5 J/cm^2^, whereas Ce6 treated cells showed 48.9% cytotoxicity. A laser irradiation of 10 J/cm^2^ resulted in 74.8% cytotoxicity for SWCNTs-HA-Ce6 treated cells, while 60.8% cytotoxicity for Ce6 treated cells. At 24 h, the percentage of cytotoxicity was significantly increased in 10 J/cm^2^ irradiated and SWCNTs-HA-Ce6 treated samples compared to 5 J/cm^2^ (*p* < 0.05) and 0 J/cm^2^ (*p* < 0.01). The percentage of cytotoxicity was significantly increased in 10 J/cm^2^ irradiated and Ce6 treated samples compared to 0 J/cm^2^ (*p* < 0.001). Similarly, the cytotoxicity was significantly increased in 5 J/cm^2^ irradiated and Ce6 treated samples compared to 0 J/cm^2^ (*p* < 0.01). There were no statistically significant differences observed in 0 J/cm^2^ irradiated and SWCNTs-HA-Ce6 treated samples compared to 5 J/cm^2^ (*p* = 0.059); 5 J/cm^2^ irradiated and Ce6 treated samples compared to 10 J/cm^2^ (*p* = 0.051).

The 48 h cytotoxicity assay results are shown in Figure 9, the observed results revealed that the non-irradiated 0 J/cm^2^ showed lower percentage of cytotoxic behavior for Ce6 (32.79%) and SWCNTs-HA-Ce6 (35.59%). The results of laser irradiation at 5 J/cm^2^, revealed that the SWCNTs-HA-Ce6 treated cells showed the cytotoxicity of 71.85%, whereas the percentage cytotoxicity for Ce6 was 60.9%. The laser irradiation results at 10 J/cm^2^, revealed that the SWCNTs-HA-Ce6 treated cells showed the cytotoxicity of 86.7%, whereas the percentage cytotoxicity for Ce6 was 67.7%. At 48 h, the percentage of cytotoxicity was significantly increased in 10 J/cm^2^ irradiated and SWCNTs-HA-Ce6 treated samples compared to 0 J/cm^2^ (*p* < 0.01); similarly, the cytotoxicity was significantly increased in 5 J/cm^2^ irradiated and SWCNTs-HA-Ce6 treated samples compared to 0 J/cm^2^ (*p* < 0.01). The percentage of cytotoxicity was significantly increased in 10 J/cm^2^ irradiated and Ce6 treated samples compared to 0 J/cm^2^ (*p* < 0.05). There were no statistically significant differences observed in 0 J/cm^2^ irradiated and SWCNTs-HA-Ce6 treated samples compared to 5 J/cm^2^ (*p* = 0.084), 5 J/cm^2^ irradiated and Ce6 treated samples compared to 10 J/cm^2^ (*p* = 0.476), and 5 J/cm^2^ irradiated and SWCNTs-HA-Ce6 treated samples compared to 10 J/cm^2^ (*p* = 0.117).

Cytotoxicity results are one of the most important parameters for synthesized compounds that are intended for the treatment of cancer. The increased percentages of cytotoxicity observed during 48 h at a dose of 10 J/cm^2^ are indicative of increased bioavailability, uptake profile, and biological activity of Ce6 released from SWCNTs-HA-Ce6. SWCNTs-HA-Ce6 exhibited greater cytotoxicity at both 24 and 48 h compared with Ce6 alone in both 5 and 10 J/cm^2^.

#### 3.3.3. Annexin V–Fluorescein Isothiocyanate (FITC) and Propidium Iodide (PI) Assay Using Flow Cytometry

In PDT, the mechanism of cell death, which directly destroys cancer cells through apoptosis or necrosis [31], is well established. The dual staining with Annexin V-FITC and PI effectively distinguished between early apoptosis (EA) and late apoptosis (LA). Annexin V binds phosphatidylserine (PS), which translocates to the outer leaflet of the plasma membrane during EA, a hallmark of apoptotic progression while maintaining membrane integrity. Propidium iodide (PI), in contrast, penetrates only cells with compromised membrane integrity, marking cells in LA or necrosis. This dual staining allows for precise differentiation between cells in the initial stages of apoptosis and those undergoing advanced apoptosis or secondary necrosis.

**Figure 10:**
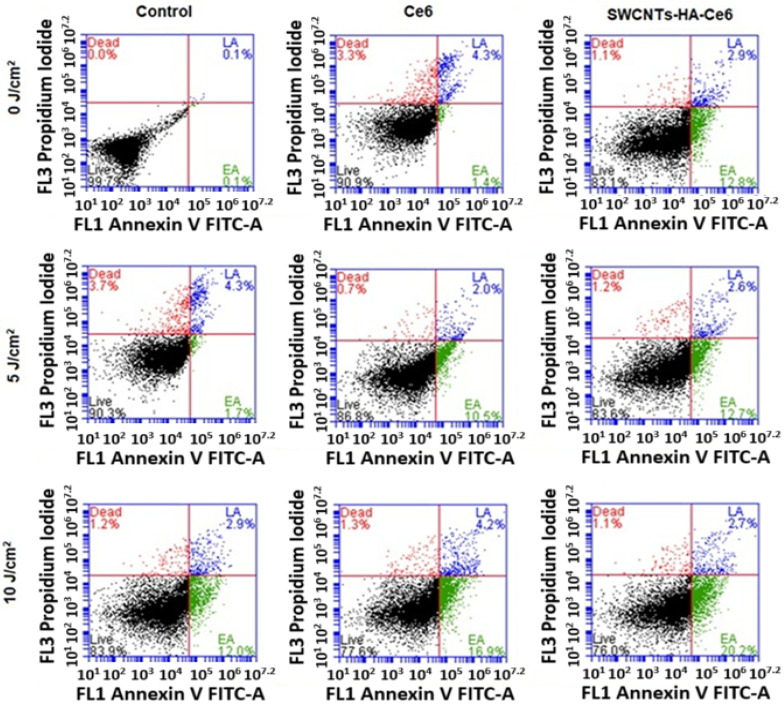
Flow cytometry analysis using Annexin V PI for cell death, laser irradiated at 660 nm of fluence 0, 5, and 10 J/cm^2^ of Ce6, SWCNTs-HA-Ce6 on CCSCs incubated for 24 h, where Live cells (Black) and EA (Green) stand for early apoptosis, LA (Blue) stands for late apoptosis, and Dead (Red) stands for necrosis.

**Figure 11:**
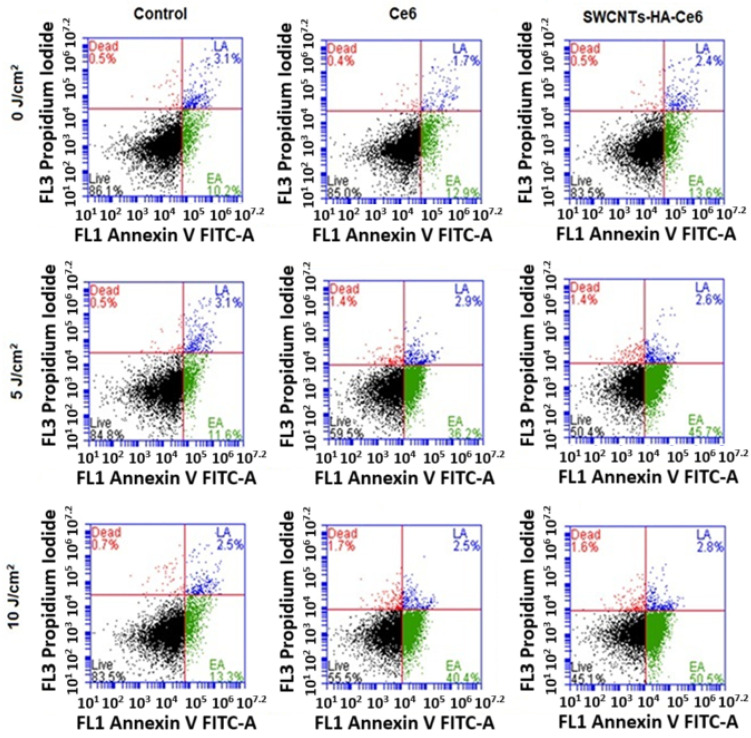
Flow cytometry analysis using Annexin V PI for cell death, laser irradiated at 660 nm of fluence 0, 5, and 10 J/cm^2^ of Ce6, SWCNTs-HA-Ce6 on CCSCs incubated for 48 h, where Live cells (Black), EA (Green) stands for early apoptosis, LA (Blue) stands for late apoptosis, and Dead (Red) stands for necrosis.

From the literature, EA is characterized by cellular shrinkage, chromatin condensation, and PS externalization without loss of membrane integrity. In contrast, LA involves further progression with loss of membrane integrity, DNA fragmentation, and the eventual breakdown of cellular structures. These distinctions are critical for understanding the effects of therapeutic interventions on apoptotic pathways and were central to the analysis conducted in this study [32]. In photodynamic effects, ROS are formed, which cause cell death by oxidizing and degrading cellular components. Flow cytometry was used to assess the cell death mechanism of PDT in isolated CCSCs. CCSCs were cultured in small Petri dishes, incubated with Ce6 and SWCNTs-Ce6-HA, and then treated with 0, 5, and 10 J/cm^2^ at 660 nm. Cells were incubated 24 and 48 h after treatment and evaluated in this study. A flow cytometry analysis pattern is presented in Figure 10 and Figure 11. The percentages of apoptotic cells in the treated groups are comparatively higher than those in the control groups. A higher percentage of cells in the early and late apoptosis quadrants cause cell death.

At 24 h (Figure 10), the SWCNTs-HA-Ce6 treated cells with no laser irradiation (0 J/cm^2^) showed that the percentage of EA and LA was about 12.8 and 2.9% whereas the Ce6 treated EA and LA was about 1.4 and 4.3%. The SWCNTs-HA-Ce6 treated cells with the 5 J/cm^2^ laser fluence showed that the percentage of EA and LA was about 12.7 and 2.6% whereas the Ce6 treated EA and LA is about 10.5 and 2.0%. The SWCNTs-HA-Ce6 treated cells with 10 J/cm^2^ laser fluence showed that the percentage of EA and LA is about 20.2 and 2.7% whereas the Ce6 treated EA and LA was about 16.9 and 4.2%. %. The Ce6 treated samples showed fewer EA and LA cells in 0, 5, and 10 J/cm^2^ irradiated samples compared to SWCNTs-HA-Ce6. The results of SWCNTs-HA-Ce6 treated and 10 J/cm^2^ irradiated samples showed the prominent apoptotic effects to the CCSCs compared to the control.

At 48 h (Figure 11), the SWCNTs-HA-Ce6 treated cells with no laser irradiation (0 J/cm^2^) showed that the percentage of EA and LA was about 13.6 and 2.4% whereas the Ce6 treated EA and LA is about 12.9 and 1.7%. The SWCNTs-HA-Ce6 treated cells with the 5 J/cm^2^ laser fluence showed that the percentage of EA and LA was about 45.7 and 2.6% whereas the Ce6 treated EA and LA is about 36.2 and 2.9%. The SWCNTs-HA-Ce6 treated cells with 10 J/cm^2^ laser fluence showed that the percentage of EA and LA was about 50.5 and 2.8% whereas the Ce6 treated EA and LA was about 40.4 and 2.5%. The Ce6 treated samples showed fewer EA and LA cells in 0, 5, and 10 J/cm^2^ irradiated samples than SWCNTs-HA-Ce6. The results of SWCNTs-HA-Ce6 treated and 10 J/cm^2^ irradiated samples showed the prominent apoptotic effects to the CCSCs compared to the control.

#### 3.3.4. Oxidative Stress Visualization Immediately Following Laser Irradiation

There are two types of PDT mechanisms, type I and type II, in a type I reaction, the excited PS interacts directly with a substrate, such as a cell membrane or molecule, leading to hydrogen atom abstraction or electron transfer. This generates free radicals and radical ions, which react with molecular oxygen to form reactive oxygen species (ROS) like O_2_^−^, HO, and H_2_O_2_, causing oxidative damage and biological lesions. However, in PDT, the Type II reaction is predominant, where singlet oxygen is the primary cytotoxic agent responsible for biological effects, both based on ROS production that leads to cellular damage or death following laser irradiation [33]. SWCNTs-HA-Ce6 was treated at 0, 5, and 10 J/cm^2^ fluence on Petri dishes containing glass cover slips and CCSCs were cultured in these dishes.

**Figure 12:**
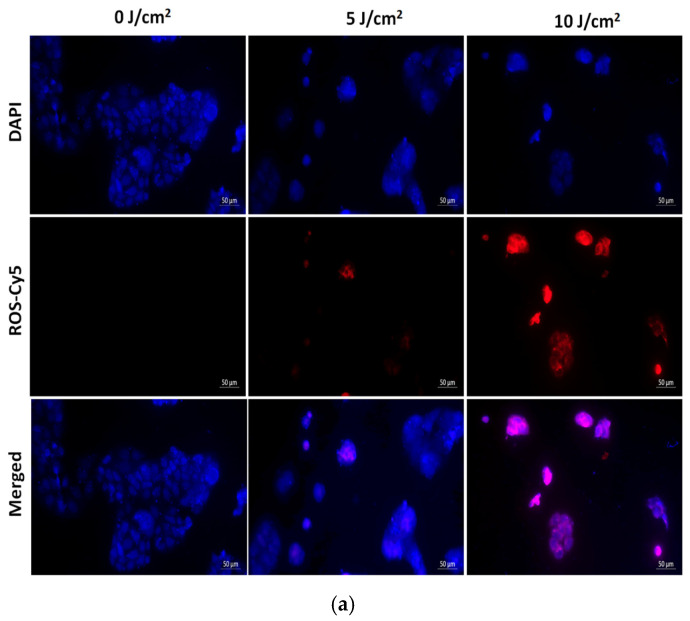

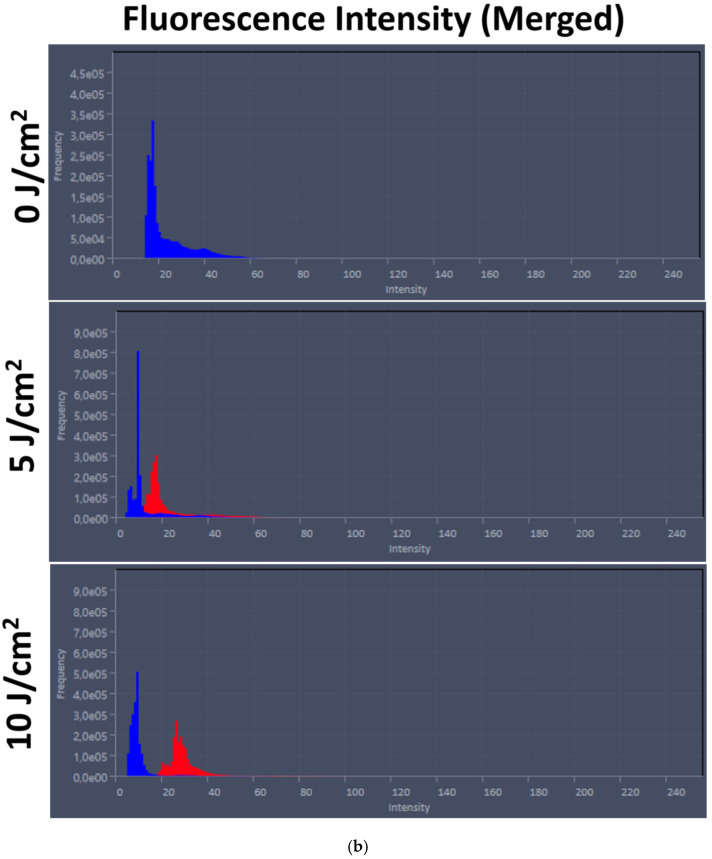
(**a**): Detection of oxidative stress on CCSCs using intracellular ROS kit. CCSCs are laser irradiation at 660 nm of fluence 0, 5, and 10 J/cm^2^ of Ce6, SWCNTs-HA-Ce6 images are represented in graph. Counter-stained using DAPI. Scale bar 50 µm. (**b**): Detection of oxidative stress on CCSCs using intracellular ROS kit. CCSCs are laser irradiation at 660 nm of fluence 0, 5, and 10 J/cm^2^ of Ce6, SWCNTs-HA-Ce6 and fluorescence intensity of the merged images are represented in graph. Counter-stained using DAPI. Cy5—Red color fluorescence; DAPI—Blue color fluorescence.

CCSCs were incubated for one hour with a fluorometric intracellular reactive oxygen species kit, and ROS production was analyzed under a fluorescent microscope with DAPI used as nuclei counterstaining. Figure 12a,b illustrates the captured images, demonstrating that 0 J/cm^2^ did not produce any red fluorescence due to the absence of ROS production. When laser irradiated at 5 J/cm^2^ and 10 J/cm^2^ fluences, red fluorescence was observed in different ranges and was measured as fluorescence intensity. There was a reduction in ROS production at 5 J/cm^2^ compared with 10 J/cm^2^, while the ROS intensity increased with an increase in fluence at 660 nm.

## 4. Conclusions

An in vitro study of the synthesized nanobiocomposite was successfully conducted using CCSCs. It has shown positive results in targeting CCSCs and causing pronounced cell death. Several factors contribute to the performance of PDT, including singlet oxygen release, absorbed light and its depth of penetration into the cell, as well as molecular stability and the allocation of PS [34]. It has recently been reported that CSCs were detected in many solid tumors and that they may be responsible for radiotherapy and metastatic potential, recurrence, and resistance to chemotherapy. According to many studies, solid tumors contain a small number of cells that can self-renew, proliferate infrequently, and initiate or metastasize [35]. In cancer therapy, the current treatment methodologies are prone to destroy only tumor cells and may fail to eradicate CSCs; therefore, new therapies are necessary to target and destroy CSCs and to eradicate the tumor. In our research, CCSCs were successfully isolated from colon cancer cell line (Caco-2). Then, the presence of isolated CCSCs were confirmed by using different characterization techniques that include immunofluorescence, flow cytometry, and microscopy to observe the formation of spheroidal morphology in CCSCs.

The synthesized SWCNTs-HA-Ce6 molecules successfully targeted the CCSCs and released the Ce6 molecule, which caused the CCSCs to be destroyed. In CCSCs, cell surface markers such as CD133, CD24, and CD44 are overexpressed, and the functionalized HA on SWCNTs targets CD44 receptors. The CCSCs were irradiated at 660 nm in two different laser fluences (5 and 10 J/cm^2^) were studied to confirm the efficacy of SWCNTs-HA-Ce6. In comparison with 5 J/cm^2^, CCSCs were observed to exhibit better cytotoxic behavior when they were irradiated with 10 J/cm^2^. The cells treated with 10 J/cm^2^ displayed a higher percentage of cell death, higher apoptotic activity, and more prominent nuclear damage than the cells treated with 5 J/cm^2^. In the present study, a novel nanocarrier, SWCNTs-HA-Ce6, was demonstrated as an effective tool for targeting and eliminating CCSCs. According to these results, SWCNTs-HA-Ce6 has the potential to be a more promising material for the treatment of colon cancer through PDT. To advance the clinical applicability of this nanocomposite, future research should focus on comprehensive biocompatibility and toxicity assessments, detailed pharmacokinetic and pharmacodynamic evaluations, optimization of drug delivery efficiency, and rigorous preclinical investigations using animal models. These studies will be crucial in ensuring the safety, efficacy, and translational potential of this nanoplatform for clinical treatment.

## Figures and Tables

**Table 1:** Laser parameters.

Parameters	
Laser type	Semiconductor diode
Wavelength (nm)	660
Emission	Continuous wave
Power output	104 mW
Power density	11.45 mW/cm^2^
Fluences (J/cm^2^)	5 and 10
Irradiation time	7 min 16 s for 5 J/cm^2^ 14 min 32 s for 10 J/cm^2^
Photosensitizer	Chlorin e6
Height of laser	8 cm

## Data Availability

The raw data supporting the conclusions of this article will be made available by the authors on request.
